# Quality of care and health insurance uptake in Namibia

**DOI:** 10.4102/jphia.v17i1.1431

**Published:** 2026-05-21

**Authors:** Nigel James, Yubraj Acharya, Ndilimeke JC Nashandi, Stephen A. Matthews, Yosef Bodovski

**Affiliations:** 1 Department of Health Studies, School of Arts and Sciences, University of Richmond, Richmond, United States; 2 Department of Health Policy and Administration, Faculty of Health and Human Development, The Pennsylvania State University, Pennsylvania, United States; 3 Department of Psychology and Social Work, Faculty of Allied Health, University of Namibia, Windhoek, Namibia; 4 Department of Sociology, Anthropology and Demography, The Pennsylvania State University, Pennsylvania, United States; 5 Population Research Institute, The Pennsylvania State University, Pennsylvania, United States

**Keywords:** health insurance, antenatal care, quality of care, spatial analysis, universal health coverage, Namibia

## Abstract

**Background:**

Despite efforts to expand health insurance in low- and middle-income countries, uptake remains low. Poor quality of care, especially in public facilities, is often cited as a barrier to health insurance enrolment but limited empirical evidence exists.

**Aim:**

We examine whether the quality of antenatal care (ANC) influences women’s enrolment in health insurance programmes in Namibia.

**Setting:**

In Namibia, insurance uptake is modest but growing, and health facility density and ANC quality vary regionally.

**Methods:**

We link Namibia’s 2009 Service Provision Assessment (SPA), a census of all health facilities, with the 2013 Demographic and Health Survey (DHS). Using geospatial data, we construct a cluster-level ANC quality index covering structural readiness, process adherence and client experience. Multivariate regressions estimate the association between ANC quality and insurance uptake, adjusting for socioeconomic and demographic factors.

**Results:**

We find no statistically significant association between ANC quality and insurance uptake nationally. However, the relationship varies by region: in areas with low health facility density (south), higher ANC quality is marginally associated with increased insurance uptake, while no significant association is found in regions with greater facility density (north). Indicators of wealth and education consistently predict higher enrolment, regardless of ANC quality.

**Conclusion:**

Improving ANC quality is essential for health outcomes but, on its own, is unlikely to drive insurance uptake nationally. Quality appears more influential in underserved regions, while socioeconomic inequalities dominate enrolment patterns.

**Contribution:**

This study expands scarce low- and middle-income countries (LMICs) evidence on quality and insurance demand, applies a novel geospatially linked ANC quality index using a facility census and highlights the need to tailor insurance expansion strategies to subnational contexts of socioeconomic inequality and service access.

## Introduction

Achieving universal health coverage (UHC) is an important policy goal in both high- and low-income countries. Expanding health insurance to cover more people, increasing the scope of healthcare services and offering financial protection are among the policy tools used to advance this goal.^1,2^ Many low- and middle-income countries (LMICs) are promoting health insurance schemes to shield individuals from the financial burden of healthcare and the risk of catastrophic health expenditures (CHEs) – out-of-pocket payments so high relative to income that they push families into poverty or crowd out basic needs.^3,4,5^ Despite sustained efforts, however, enrolment in LMICs remains low.^6,7^ This is concerning, as health insurance programmes are designed not only to shield families from CHEs but also to reduce their vulnerability to poverty – an overarching target of the Sustainable Development Goals (SDGs).

Prior work identifies drivers of low enrolment on both the demand and supply sides. On the demand side, inability to pay premiums and incomplete understanding of insurance as a risk management mechanism frequently depress participation.^7,8,9,10^ On the supply side, the predominance of informal employment in many LMICs constrains contribution collection and risk pooling.^7,8,11^ Another widely discussed factor is the poor quality of care in public facilities, which has been argued to deter enrolment.^12,13,14,15^ Following Donabedian, we define quality as a combination of structure (resources and infrastructure), process (adherence to clinical protocols) and experience (patients’ perceptions and satisfaction).^16^ The hypothesis is straightforward: poor-quality care is thought to deter enrolment, as individuals may see little value in paying for insurance that entitles them to substandard services. Yet, despite the prominence of this assumption, the empirical evidence linking measured quality to insurance participation in LMICs remains scarce.

Most existing studies focus on the reverse relationship – namely, the impact of insurance on quality of care. In high-income countries, insurance expansion has been shown to improve access and outcomes: for example, Nabi et al. demonstrate that uninsured or publicly insured cancer patients in the United States are less likely to reach high-volume hospitals,^17^ while McWilliams summarises evidence that lack of coverage worsens access and health outcomes across multiple conditions.^18^ In LMICs, community-based schemes have shown similar effects: Michielsen et al. find that insurance in India improved accountability and strengthened patient bargaining power at the point of care, although challenges remained in ensuring service quality.^19^ Far fewer studies test whether quality influences enrolment. Evidence to date is mixed: in Ghana, lower perceived quality correlated with reduced enrolment, particularly among women,^20^ while in Ethiopia, improvements in technical quality and patient satisfaction under community-based health insurance (CBHI) did not translate into higher enrolment or renewal.^21^The lack of robust, comparable measures of quality helps explain why the role of quality in insurance enrolment remains underexplored in LMICs.^22,23^

In this study, we assess whether ANC quality is associated with women’s enrolment in health insurance in Namibia. We draw health facility information from Namibia’s 2009 Service Provision Assessment (SPA) – notably a census of all health facilities – and household information from the 2013 Namibia Demographic and Health Survey (NDHS). Using geospatial data, we link household clusters to nearby facilities and analyse the relationship between enrolment and ANC quality in a regression framework that adjusts for key socioeconomic and demographic covariates.

We chose ANC as a proxy for maternal healthcare quality for three reasons. Firstly, while maternal or infant mortality and morbidity could in principle reflect quality, these outcomes are lagging and shaped by multiple determinants beyond facility care (e.g. nutrition, transport access, emergency obstetric care, postnatal practices).^24^ As such, they are not well suited for isolating facility performance in DHS household-linked analyses. In contrast, ANC provides contemporaneous, service-specific indicators across structure, process and experience. Secondly, the ANC module in Namibia’s 2009 SPA survey is among the most comprehensive and complete sections, with detailed measures of structural readiness, provider adherence and client feedback. Thirdly, this combination of completeness, relevance to women’s health and inclusion of patient experience allows the construction of a multidimensional quality index, making ANC a particularly robust and policy-relevant proxy for examining whether measured care quality influences insurance enrolment.

Our contributions are twofold. Substantively, this is the first national analysis in Namibia to assess whether measured service quality shapes health insurance enrolment. Methodologically, the study advances prior work by constructing a multidimensional ANC quality index (structure, process and experience) and linking a facility census to a nationally representative household survey via geospatial methods. This approach moves beyond perceptions, aligns local service environments with population data and provides a replicable framework for other LMIC setting.

We focus on Namibia for three reasons. Firstly, the 2009 SPA is a facility census, enabling comprehensive national mapping of service readiness and ANC quality – rare in LMICs.^25^ Secondly, Namibia has relatively high but unequal health insurance coverage in the region, providing informative variation for studying determinants of participation.^26^ Thirdly, the temporal ordering (SPA preceding NDHS) aligns with our hypothesis that facility quality can influence subsequent household enrolment decisions. These features make Namibia an instructive setting for examining how measured quality relates to insurance enrolment and for developing an approach that can be replicated elsewhere. Geographically linking household survey clusters to health facility characteristics creates an opportunity to analyse how local service environments – including quality – relate to care-seeking and financial protection decisions.^27,28,29^

The remainder of the paper is organised as follows. We begin with an overview of the Namibian healthcare system, followed by a description of the data and the geospatial linkage of household insurance information to SPA facility data on ANC quality. Next, we outline the empirical strategies, encompassing both national and subnational analyses. We then present the key findings and conclude with a discussion of their implications, including study limitations, policy relevance and directions for future research.

## Overview of the Namibia healthcare system

The Namibian Ministry of Health and Social Services (MoHSS) regulates, organises and delivers healthcare to approximately 2.1 million people through 14 regional directorates and 34 health districts.^30,31^ Specifically, service delivery is pluralistic: the public sector provides 70% – 75% of care, faith-based institutions, 15% – 20% and the private sector, roughly 5%.^32^ Public facilities primarily serve unemployed and lower-income groups,^33^ while private providers are concentrated in the 13 cities and 26 towns; faith-based providers are especially important in rural areas.

Despite this mix, insurance enrolment remains low: > 80% of women and men aged 15–49 years remain uninsured.^31,34^ Public services are provided through 30 district hospitals, 44 health centres and 269 clinics, supplemented by roughly 1150 outreach clinics. Namibia is sparsely populated (2.4 persons per km^2^), with one in five residents living in the capital, Windhoek.^35^ While low density complicates service delivery in remote areas, coverage disparities are driven more by socioeconomic than purely geographic factors.

Government health spending constituted 13% of the national budget in 2015 (near the Abuja 15% target).^36,37^ Financing shares of total health expenditure (THE) were 64% government, 20% employers, 10% households and 6% donors.^36,38^ Household spending is largely out of pocket, and approximately 71% of government health expenditure is directed to secondary or tertiary care.^38^

The private sector is financed mainly through medical-aid funds – either open (available to all) or closed (restricted to firms or industries).^34,38^ The government operates the Public Service Medical-Aid Scheme (PSEMAS), a large, closed scheme. In this voluntary programme, employees contribute a flat rate and the government covers shortfalls; because contributions are not income related and benefits accrue disproportionately to higher earners, PSEMAS functions as a regressive scheme and imposes high fiscal costs.^39^

Policy debates have focused on stronger risk pooling and equity, including proposals for a Namibia Medical Benefits Fund (NMBF) as a social health insurance (SHI) scheme, alongside broader hybrid models to enhance efficiency and financial protection.^32,40,41^ Recent developments underline the centrality of public-sector quality and confidence: in 2025, authorities announced that beginning in April 2026, senior officials would be required to use public hospitals and clinics, a confidence-building measure paired with planned upgrades in staffing, medicines and infrastructure.^42^

Health insurance enrolment is highly unequal. Only 5% of individuals in the poorest consumption quintile are covered versus 70% in the wealthiest quintile.^43,44^ Coverage is also higher among urban residents and those with higher education.^31,45^ In 2014, private insurance holders (including dependents and pensioners) contributed roughly Namibian (N) $2.5 billion ($220m) in premiums.^46^ High unemployment and a large informal sector (about 1.53 million people were unemployed, economically inactive or under 15 in 2015^45^) limit participation in voluntary schemes and heighten vulnerability to catastrophic health costs.

ANC service coverage in Namibia is high – nearly universal for a first antenatal visit and roughly two-thirds for the recommended fourth visit – yet quality is uneven.^31^ Analyses of Namibia’s SPA data by Do et al. show that while structural readiness (equipment, diagnostics and staffing) is generally adequate, there is wide variation in process quality and client experience, with inconsistent counselling and protocol adherence.^47^ Global benchmarks, similarly, highlight that across LMICs, high ANC utilisation often fails to translate into consistent quality, with deficits in provider training, diagnostic supplies and counselling services.^24^

Regionally, Namibia’s coverage of the first ANC visit is among the highest (~99%).^48^ Malawi, Zambia and Zimbabwe also report high coverage, generally in the mid- to high-90% range.^49^ Yet across these settings, quality gaps are evident: fewer women receive key evidence-based components such as blood pressure checks, tetanus vaccination or nutrition counselling.^47,50,51^ Facility assessments in selected districts of Malawi, Zambia and Zimbabwe reinforce this point, showing that fewer than half of facilities met basic ANC infrastructure standards and that process quality (e.g. counselling, blood or urine tests) was frequently suboptimal.^50^ Analyses of SPA data in Namibia, Kenya and Senegal similarly reveal deficiencies in infrastructure and supplies, prolonged waiting times and inconsistent adherence to ANC protocols. Importantly, higher-level facilities do not consistently exceed clinics in process quality, and waiting time emerges as a robust predictor of lower client satisfaction.^49^ Thus, Namibia pairs unusually high ANC coverage with persistent process and counselling gaps – a pattern broadly seen across the region – making it a particularly informative setting to test whether measured ANC quality is associated with women’s insurance enrolment.

Health insurance affordability remains a salient challenge. From 2009 to 2013, average monthly medical-aid premiums increased to approximately N$1633.00, including a 17.2% rise in 2010 relative to 2009.^33,52^ During this period, the average household earned about N$7500.00 per month, implying that premiums represented roughly 22% of income, with an even heavier burden on poorer households.^53^ By 2023, premiums ranged from N$756.00 for an entry-level plan to N$3742.00 for a top plan.^54^ With gross national income (GNI) per capita estimated at the United States (US) $4870.00 (about N$6087.00 per month), the top plan accounted for roughly 61.5% of average monthly income, while the entry plan still required 12.4%.^55^ This financial reality demonstrates that despite economic changes, healthcare insurance premiums continue to impose a significant financial strain on many households, particularly those earning below the national average, and especially those in poverty. Although maternal and child health services are subsidised in public facilities, women face indirect costs (transport, supplies and accommodation near facilities). In rural areas, maternity waiting homes mitigate travel but charge daily fees.^56^ Accordingly, even where services are subsidised, insurance can protect against indirect and ancillary costs.^5,57^

Understanding the broad determinants of insurance enrolment – including ANC quality – is therefore of high policy importance.

## Research methods and design

### Data

We combine two nationally representative surveys from Namibia: the 2013 Demographic and Health Survey (NDHS) and the 2009 SPA survey.^31,58^ The NDHS provides data on women’s health insurance status and socio-demographic characteristics (e.g. age, household size, marital status, education and wealth). The SPA provides information on the quality of ANC services at all health facilities delivering ANC services.

#### The 2013 Namibia demographic and health survey

The NDHS collects data approximately every 5 years from a nationally representative sample of households. The 2013 survey provides estimates of demographic and health-related indicators for both rural and urban areas in the 13 administrative provinces of Namibia. The survey used a two-stage cluster sampling design. In the first stage, 554 NDHS clusters – 269 urban and 285 rural – were selected with probability proportional to their population size from a national sample frame. A complete household listing and mapping exercise was conducted in all selected clusters. In the second stage, a fixed number of 20 households were selected in every cluster according to equal probability systematic sampling. All women aged 15–49 years in the sampled households were eligible for individual interviews. Of the 9940 women eligible for interview, 9176 were successfully interviewed.

To ensure confidentiality, the cluster coordinates were checked and geographically displaced before the geographic dataset was publicised.^27,28,29,31,59^ Urban cluster coordinates were displaced by a maximum of 2 km, while those in rural areas were displaced by up to 5 km. Additionally, 1% of randomly selected rural clusters were displaced by up to 10 km.

In this study, we conduct both descriptive and regression modelling analyses. We present descriptive sample characteristics in this Methods section to contextualise the analytic dataset; inferential results are reported in the Results section. For our analysis, we use the Individual Recode file, which has information on 3822 women aged 15–49 years who attended ANC services during the 5 years preceding the survey. These women were from 550 NDHS clusters with global positioning system (GPS) data. Table 1 shows their background characteristics.

**TABLE 1:** Descriptive statistics for the demographic and health survey sample of households (*N* = 3822).

Variables	Mean	Frequency	s.d.
**Mothers’ characteristics**			
Insured	0.13	-	-
Education (years)	8.50	-	3.63
Employed	0.43	3803	-
**Marital status**			
Never married	0.47	1804	-
Currently married	0.47	1805	-
Formerly married	0.06	213	-
Institutional delivery	0.89	-	-
**Birth order number**			
1	0.32	1224	-
2–3	0.42	1603	-
4–5	0.17	639	-
≥ 6	0.19	356	-
Household head age	43.83	-	16.18
Household size	6.27	-	3.56
**Wealth status**			
Poorest	0.20	743	-
Poorer	0.21	807	-
Middle	0.22	853	-
Richer	0.22	853	-
Richest	0.14	546	-
Urban	0.48	-	-
**Province**			
Caprivi	0.09	323	-
Erongo	0.08	303	-
Hardap	0.07	253	-
Karas	0.08	296	-
Kavango	0.10	382	-
Khomas	0.09	330	-
Kunene	0.08	287	-
Ohangwane	0.09	335	-
Omaheke	0.06	228	-
Omusati	0.07	282	-
Oshana	0.06	228	-
Oshikoto	0.07	263	-
Otjozondjupa	0.08	290	-

*Note:* This table summarises key characteristics of the 2013 Namibia DHS sample, including maternal demographics, household composition and regional distribution. Values shown are means/proportions, frequencies and s.d. where applicable.

s.d., standard deviation.

Thirteen per cent of the sample has health insurance. The mean education is 8.5 years. Household heads’ average age and household size are 43.8 years and 6.7, respectively. The household wealth distribution is categorised into five quintiles, with the richest constituting 14% and the poorest 20%. Wealth is based on the DHS household asset index and divided into quintiles (five equal groups from poorest to richest). Urban households make up 48% of the sample, with Kavango, Khomas and Ohangwena representing the most populous provinces.

#### The 2009 Namibia service provision assessment

**FIGURE 1:**
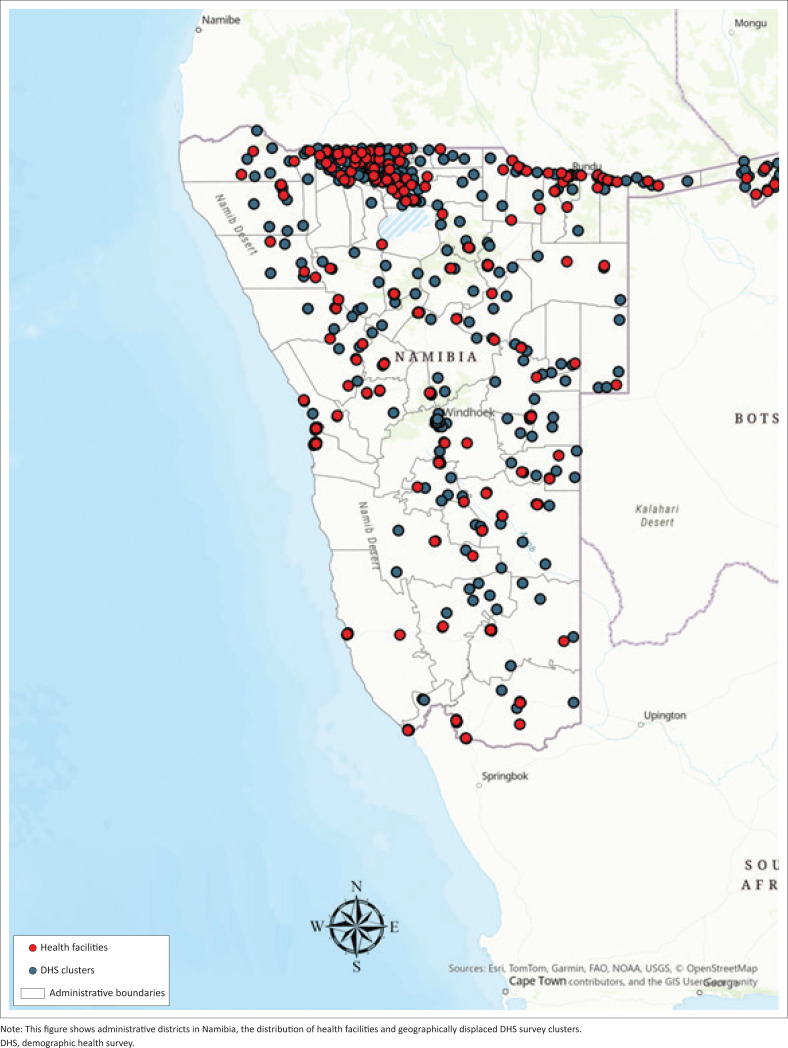
Health facility, clusters and regional boundaries in Namibia.

To complement household-level data from the NDHS, we draw facility-level measures from the 2009 SPA, a census of all health facilities in Namibia. The SPA is a cross-sectional survey that collects health facility-level data on the quality of healthcare services, health infrastructure and training and incentives provided to health staff.^60^ It contains four modules: (1) Facility Inventory, covering availability of services, infrastructure, medical sundries and health workers and staff training needs. (2) Provider Interviews, covering health workers’ qualifications, experience and perceptions of service delivery. (3) Patient Observations, measuring the extent to which health staff adheres to service delivery and treatment protocols and guidelines. (4) Client Satisfaction, measuring clients’ understanding of services and general perception of the quality of services received. The Namibia 2009 SPA survey is based on a census of all health facilities in the country – 411 facilities. Unlike the DHS data, facilities’ coordinates are not displaced. Figure 1 shows the location of DHS clusters (displaced) and SPA facilities.

Our main analytic sample of health facilities comprises 263 ANC facilities. We construct a facility’s ANC quality score primarily using the inventory, the health provider interview and client satisfaction questionnaires (see Online Appendix 1 – Table 2-A1). Overall, we include health facilities if they reported providing ANC services, completed the ANC portion of the facility audit and had non-missing data across the three questionnaires. Most of these facilities are clinics, accounting for 81% of the sample, followed by health centres at 16% and hospitals at 2%. A significant proportion (89%) of the facilities are publicly managed, while 9% are under mission and/or NGO entities, and only 2% are privately managed. The highest concentration of ANC facilities is found in Kavango (17%), followed by Ohangwena and Kunene (10% each). Khomas has the lowest representation, accounting for only 1% of the sample. Online Appendix 1 – Table 1-A1 shows the distribution of facilities offering ANC services by type, managing authority and location.

The raw mean facility ANC quality score is 73.7%, with a standard deviation (s.d.) of 8.5. Facilities with the lowest scores (42% – 57%) are primarily located in Zambezi (formerly Caprivi), Khomas and Otjozondjupa. Conversely, facilities in Ohangwena and Omusati achieve scores above 90%. Kavango exhibits a wide range, encompassing both low- and high-performing facilities. These quality patterns align with findings from other studies and government reports examining ANC quality across different regions in Namibia.^25,61^

### Measures

#### Distance-based accessibility

According to WHO guidelines and various studies on healthcare access, acceptable distances to health facilities range from 5 km to 15 km,^62,63^ with an optimal benchmark set at 5 km.^64^ In our sample, 59% of clusters (2384 women) are within 10 km of a health facility offering ANC. Expanding the radius to 15 km includes 65% of clusters (2708 women). About 30% of women (1114) reside beyond 15 km, suggesting potential distance barriers in accessing ANC services, regardless of other quality dimensions. As a supplementary check, we include a 30 km buffer to compare outcomes across broader catchments. The density of ANC facilities varies widely, with fewer options in remote areas.

We also construct a binary variable from DHS data capturing women’s perceived distance barriers: assigned ‘0’ if distance is reported as a problem and ‘1’ if not. In our sample, the proportion perceiving distance as a barrier increases with actual distance. This aligns with prior evidence that distance not only affects utilisation but also shapes perceptions of quality and accessibility.^65^

#### Weighted antenatal care quality index

To operationalise quality of care, we follow Donabedian’s framework and incorporate structural, process and experience indicators. Structural indicators (maximum 23 points) include availability and use of diagnostic and anthropometric tools (sphygmomanometers, height boards and test kits) and referral systems. Process indicators (maximum 29 points) cover examinations, history taking, counselling on pregnancy, institutional delivery, iron folate supplementation, vaccinations and inquiry about danger signs. Experience indicators (maximum 10 points) capture patient-reported feedback on care received. All indicators are binary and summed using weights (Online Appendix 1 – Table 2-A1), producing a facility-level score scaled 0–100. Online Appendix – Figure 1-A1 to Online Appendix – Figure 3-A1 show the distribution of weighted quality scores at 10 km, 15 km and 30 km buffers.

#### Insurance status

The main outcome is a binary variable for whether a woman had any type of insurance in 2013. In the NDHS, women were asked if they were covered by health insurance, with a ‘yes or no’ response. Insurance types include mutual funds, employer-based, private or commercial and government schemes. We focus on women’s insurance status because ANC is a service specific to women, and our quality index is constructed from ANC data, making this alignment between exposure and outcome most appropriate.

#### Other covariates

We adjust for demographic and socioeconomic characteristics, including education (primary, secondary, college, graduate), employment, marital status, parity, household size, age of the household head and the household wealth index. We also include community-level factors such as region and urban–rural residence. In addition, we control for the proportion of private facilities within each cluster, recognising that the public–private mix can influence both perceived and actual quality.^66,67,68^ Finally, we account for women’s reported perception of distance barriers.

### Linking the demographic and health survey and service provision assessment data

Conceptually, our study employs the *service area linkage* technique, a spatial analysis approach that has gained prominence with the increasing availability of geocoded survey and facility data.^27,28,59^ This method integrates facility-level characteristics with household-level survey data by geographically linking clusters to the facilities they are most likely to use, thereby creating a measure of the local health service environment. In our analysis, the 2009 ANC quality index is treated as a proxy for the healthcare environment that women encountered during antenatal visits. In most low- and middle-income settings, healthcare quality tends not to change drastically without major financial or infrastructure investments. It is therefore reasonable to interpret the 2009 index as reflecting a persistent healthcare environment rather than a single-year snapshot. A detailed discussion of the theoretical foundations of this method is provided elsewhere^28,59^; here, we describe our implementation.

A central challenge is that DHS household cluster coordinates are geographically displaced to protect confidentiality, while SPA facility coordinates are not. This makes a naive ‘nearest facility’ assignment prone to misclassification, as displaced clusters may not correspond to the true facilities used by respondents. Prior work has emphasised this limitation and developed approaches to reduce bias.^27,29^ To address the problem, we assign ANC quality scores to clusters by averaging the scores of all government facilities within multiple buffer zones (10 km, 15 km and 30 km) around each NDHS cluster centroid. This approach captures the broader service environment rather than assuming households rely on a single facility.

**FIGURE 2:**
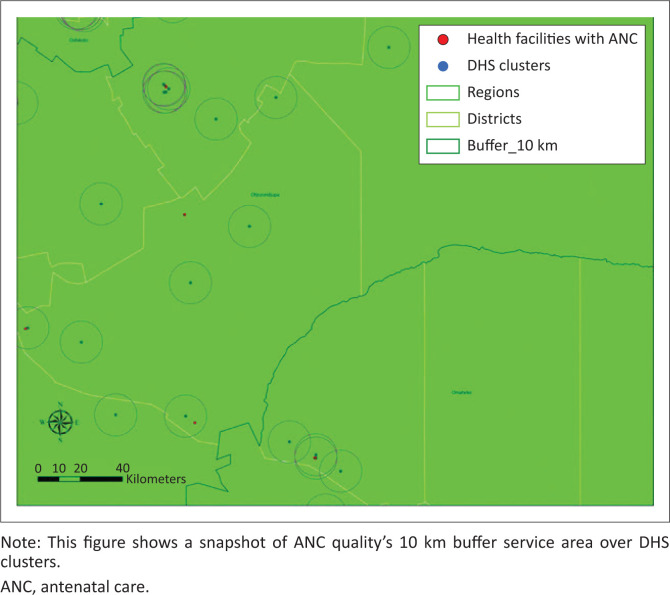
Demographic and health survey – Service provision assessment linkage based on 10 km cluster buffer.

We implemented the linkage in ArcGIS 10.8.1. Firstly, we created 10 km, 15 km and 30 km buffers around each NDHS cluster. Secondly, we applied inverse distance weighting (IDW) and spatial join-by-location techniques to generate weighted averages of ANC quality scores for all facilities within each buffer. IDW assigns greater weight to facilities closer to the cluster centroid, ensuring that nearer facilities influence the score more strongly. Thirdly, we used a join-by-table function to merge the computed cluster-level quality scores with the NDHS data. In essence, each DHS cluster is assigned an ANC quality score that reflects the weighted average service environment within the buffer radius. This method reduces bias introduced by coordinate displacement by smoothing across multiple facilities instead of relying on one ‘nearest’ match. Figure 2 illustrates the linkage process using the 10 km buffer as an example.

### Statistical analysis

We estimate the following multivariate regression model (Equation 1):


$$Ins_{ij}=\alpha +\beta_{1}ANCQ_{j}+X_{ij}^{\prime }\beta +\varepsilon_{ij}.$$ [Eqn 1]

where *Ins*_*ij*_ is the health insurance status of women *i* residing in cluster *j*; *ANCQ*_*j*_ is the average ANC quality score in facilities within ‘x’ km from the woman’s residence where x {10,15,30}. *X*_*ij*_ represents the household and community-level indicators measured in 2013. *ε*_*ij*_ is the usual error term.

The coefficient of interest, *β_1_*, captures the association between ANC quality and insurance enrolment. Consistent with prior arguments that poor-quality care may deter enrolment, we hypothesise *β*_*1*_ > 0, since higher quality should, in theory, increase perceived value of enrolment. We estimate the coefficients in the regression above using a linear probability model for ease of interpretation.

To assess robustness to functional form, we also estimated models that replaced the continuous ANC quality index with indicator variables for ANC quality quintiles (Q2–Q5, with Q1 as the reference category) at 10 km, 15 km and 30 km buffers. We further examined whether the association between ANC quality and insurance differed by urban–rural residence by including an interaction term between ANC quality and an indicator for urban clusters in the 15 km models and computing predicted probabilities from these interaction models. Finally, because health facility density is much higher in northern than southern Namibia, we estimated separate models for the north and south at the 15 km buffer and used a Wald test to assess whether the ANC quality coefficients differ between these regions. All analyses were conducted in Stata 17.0 (StataCorp, 2021),^69^ with geospatial linkages and mapping performed in ArcGIS 10.8.1.^70^

### Ethical considerations

This article followed all ethical standards for research without direct contact with human or animal subjects.

## Results

In Table 2, we report multivariate estimates from the main specification with individual- and community-level covariates. Coefficients (×100) are interpreted as percentage point (pp) changes in the probability of having insurance per one-unit increase in the ANC quality index. For the 15 km buffer (Panel A), a one-unit increase in ANC quality is associated with a 0.02 pp change in the probability of enrolment. Estimates are of similar magnitude for the 10 km buffer, and in both cases, confidence intervals include zero (i.e. not statistically significant at conventional levels).

**TABLE 2:** Coefficients from regression of insurance status on antenatal care quality.

Variables	Panel A (quality within 10 km)		Panel B (quality within 15 km)	
	Coefficient	s.e.	Coefficient	s.e.
ANC quality index	0.0001	0.0009	0.0002	0.0008
**Facility characteristics**				
Proportion of publicly managed	−0.0237	0.0266	−0.0235	0.0266
**Mothers’ characteristics**				
Education in years	0.0233**	0.0026	0.0214**	0.0023
Employed	0.1129**	0.0132	0.1105**	0.0123
**Marital status (ref never married)**				
Currently married	0.0957**	0.0153	0.0941**	0.0141
Formerly married	0.0280	0.0266	0.0255	0.0249
Institutional delivery	0.0028	0.0165	−0.005	0.0149
**Birth order (ref first)**				
2–3	−0.0228	0.0163	0.023	0.0148
4–5	0.0189	0.0232	0.0276	0.0276
≥ 6	0.0023	0.0328	0.0081	0.0294
**Household characteristics**				
Household head age	−0.0006	0.0004	−0.0006*	0.0003
Household size	−0.0166	0.0111	−0.0144	0.0103
**Wealth status (ref poorest)**				
Poorer	−0.0065	0.0164	0.0026	0.0136
Middle	0.0014	0.0190	0.0133	0.0165
Richer	0.0279	0.0216	0.0468**	0.0197
Richest	0.2426**	0.0297	0.2631**	0.0280
Urban	−0.0178	0.0166	−0.0233	0.0122
Distance is not a big problem (perceptions)	0.0164	0.0130	0.0178	0.0115
Constant	−0.1097	0.0808	−0.1101	0.0773
*R* ^2^	0.2504	-	0.2478	-
*N*	2374	-	2695	-

*Note:* This table shows coefficients (and standard errors) from a linear probability model in which we regressed insurance status on ANC quality separately at 10 km and 15 km. The regressions are modelled on a full set of covariates, which include household demographic characteristics (education level measured in years, age of household head, household size, employment and marital status, whether the birth was a home or institutional delivery and birth order), household financial characteristics (measured as a household wealth index) and community characteristics (rural versus urban and proportion of publicly managed health facilities within a given cluster and urban fixed effects).

ANC, antenatal care; s.e., standard error; ref, reference group.

* , *p* < 0.10;

** , *p* < 0.01.

To probe access-constrained settings, we re-estimated the model using a 30 km buffer (Table 3). Here, a one-unit increase in the ANC quality index is associated with a 0.07 pp change in enrolment; this estimate is also not statistically significant.

**TABLE 3:** Coefficients from regression of insurance status on antenatal care quality for 30 km buffer.

Variables	Panel A (quality within 30 km)	
	Coefficient	s.e.
ANC quality score	0.0007	0.0008
Constant	−0.1223	0.0759
*R* ^2^	0.2425	-
*N*	3053	-

Note: This table shows coefficients (and standard errors) from a linear probability model in which we regressed insurance status on ANC quality for the 30 km buffer. The regression is modelled on a full set of covariates, which include household demographic characteristics (education level measured in years, age of household head, household size, employment and marital status, whether the birth was a home or institutional delivery and birth order), household financial characteristics (measured as a household wealth index) and community characteristics (rural versus urban and proportion of publicly managed health facilities within a given cluster and urban fixed effects).

ANC, antenatal care; s.e., standard error.

A policy-relevant way to examine the association is to invert the coefficient: using the 15 km model in Table 2, an increase of roughly 50 units in the ANC quality index would be required to raise insurance coverage by 1 pp.

We also estimated a categorical specification that groups ANC quality into quintiles (Q1–Q5). Results from this sensitivity analysis (Online Appendix – Table 2-A1) mirror the continuous-index findings: relative to Q1, higher-quality quintiles show no statistically significant and no monotonic differences in enrolment at the national level.

As a further sensitivity analysis for GPS displacement, we re-estimated the 10 km and 15 km models restricting the sample to urban clusters, where DHS displaces coordinates by at most 2 km (Table 4). In these urban-only models, the ANC quality coefficients remain small and statistically non-significant, whereas education and wealth gradients are similar to those in the full sample, suggesting that the null association between ANC quality and insurance uptake is not driven by coordinate displacement.

**TABLE 4:** Coefficients from regression of insurance status on antenatal care quality for 10 km and 15 km buffers (urban clusters).

Variables	Panel A (quality within 10 km urban)		Panel A (quality within 15 km urban)	
	Coefficient	s.e.	Coefficient	s.e.
ANC quality score	0.0013	0.0013	0.0011	0.0013
Constant	−0.0771	0.1153	−0.0679	0.1173
*R* ^2^	0.2425	-	0.2685	-
*N*	1474	-	1474	-

Note: This table shows coefficients (and standard errors) from a linear probability model in which we regressed insurance status on ANC quality at 10 km and 15 km buffers, restricted to urban DHS clusters (maximum DHS GPS displacement 2 km). The regression is modelled on a full set of covariates, which include household demographic characteristics (education level measured in years, age of household head, household size, employment and marital status, whether the birth was a home or institutional delivery and birth order), household financial characteristics (measured as a household wealth index) and community characteristics (rural versus urban and proportion of publicly managed health facilities within a given cluster and urban fixed effects; the urban indicator is omitted in these models because all clusters are urban).

ANC, antenatal care; s.e., standard error.

Sensitivity analyses (Table 4) confirmed the robustness of these findings across multiple specifications, including buffer sizes, functional forms (quintiles) and urban-only subsamples.

**FIGURE 3:**
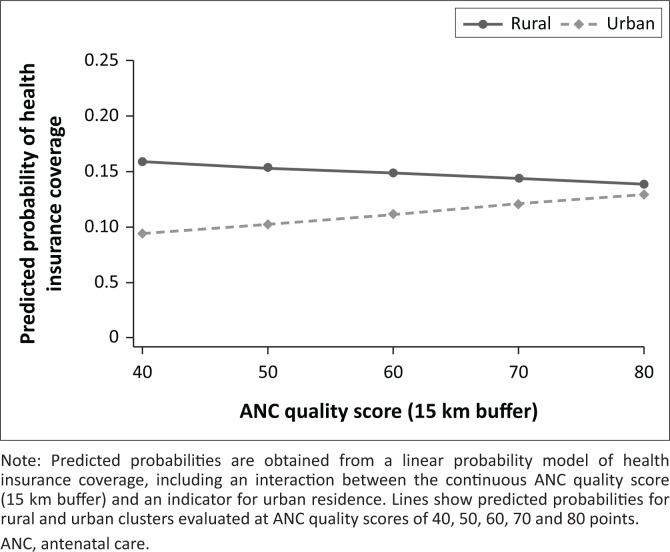
Predicted probability of health insurance coverage by antenatal care quality score and residence.

We further examined whether the association between ANC quality and insurance differed by urban–rural residence using interaction models at the 15 km buffer. Predicted probabilities from these models (Figure 3) show relatively flat quality–insurance profiles in both rural and urban areas, with only modest level differences between locations across the observed quality range. This indicates no strong evidence that ANC quality is more strongly associated with insurance uptake in either urban or rural settings.

### Regional heterogeneity

Given pronounced differences in facility density, we estimated models separately for the north (higher facility density) and south (lower density) (Table 5). In the south, a one-unit increase in the ANC quality index is associated with a 0.30 pp increase in enrolment (marginally statistically significant). In the north, the association is small and negative (−0.08 pp) and not statistically significant. A Wald test of coefficient equality confirms that the quality–enrolment association differs significantly between the north and south (Table 5).

**TABLE 5:** Heterogeneous analysis.

Variable	North		South		Test of significance
	Coefficient	s.e.	Coefficient	s.e.	
ANC quality	−0.0008	0.0009	0.0026*	0.0013	0.0350**
*R* ^2^	0.2224	-	0.2689	-	-
*N*	1504	-	1191	-	2695

*Note:* The table presents coefficients and standard errors obtained from the suest test examining the ANC quality effect on health insurance enrolment separately in the north and south at a 15 km buffer. The regressions include a comprehensive set of covariates encompassing household demographic characteristics (education level measured in years, age of household head, household size, employment and marital status, whether the birth was a home or institutional delivery and birth order), household financial characteristics (measured as a household wealth index) and community characteristics (rural versus urban, and proportion of publicly managed health facilities within a given cluster, along with urban fixed effects).

ANC, antenatal care; s.e., standard error.

* , *p* < 0.10;

** , *p* < 0.05.

**FIGURE 4:**
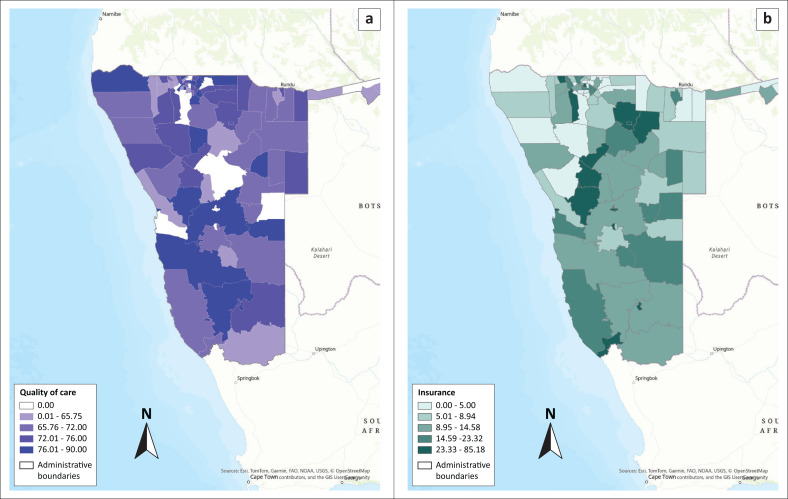
Antenatal care quality scores and health insurance coverages by administrative districts illustrating by administrative districts: (a) the percentage ANC quality scores and (b) health insurance coverages.

Figure 4 complements these results by mapping district-level ANC quality scores and health insurance coverage. The maps reveal marked spatial heterogeneity in both quality and insurance, but high-quality districts do not systematically overlap with high-insurance districts, reinforcing the regression evidence that measured ANC quality is not a primary driver of enrolment at the national scale.

### Socioeconomic gradients and interactions

**FIGURE 5:**
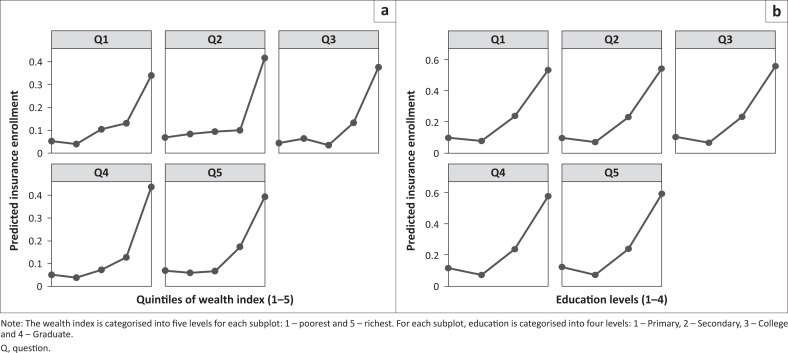
Insurance uptake by quintiles of quality quintiles across wealth and education levels (*N* = 3822) illustrating the predicted probabilities of insurance enrolment within varying quintiles of antenatal care quality (1–5) across: (a) wealth quintiles (b) education levels.

To assess whether socioeconomic status modifies the quality–enrolment relationship, we interacted ANC quality quintiles with wealth quintiles and with schooling. As shown in Figure 5, enrolment increases monotonically with wealth across all levels of ANC quality. A similar gradient is observed for education: at every ANC quality level, additional schooling is associated with higher enrolment (Figure 5). These interaction patterns, together with the main models, indicate that wealth and education are consistent, strong predictors of voluntary insurance enrolment, whereas ANC quality – whether measured continuously, in quintiles, or interacted with location – plays at most a limited role in explaining who is insured.

## Discussion

In this study, we examined whether measured quality of antenatal care (ANC) is associated with women’s enrolment in health insurance in Namibia, linking a facility census (SPA) to a nationally representative household survey (DHS). Three consistent findings emerge. Firstly, nationally, the association between ANC quality and insurance enrolment is small in magnitude and statistically non-significant across multiple buffers (10 km, 15 km and 30 km) and functional forms (continuous index and quintiles). Secondly, there is meaningful regional heterogeneity: in the south – where facility density is low – higher ANC quality is modestly and marginally associated with greater insurance uptake, while no association is detected in the more densely served north. Thirdly, socioeconomic gradients dominate: wealth and education are strong, monotonic correlates of enrolment irrespective of local ANC quality levels.

These results complement and extend the limited empirical work on whether service quality drives insurance demand in LMICs. Prior studies more often assess the reverse pathway – insurance improving resources, processes and patient satisfaction – than the demand pathway from quality to enrolment. Evidence on the influence of quality on enrolment is mixed: perception-based measures in Ghana suggest that lower perceived quality depresses participation, whereas Ethiopian CBHI evaluations indicate that observed improvements in technical quality and satisfaction did not translate into higher enrolment or renewal.^21,71^ The Namibia findings align more closely with the latter: within the ANC domain and at national scale, higher measured quality does not appear to be accompanied by higher enrolment in a substantive way.

The null findings at national level should be interpreted in light of two features of the Namibian context. Firstly, financial barriers are substantial. Premiums represent a large share of monthly income for many households, particularly in the lower quintiles; high unemployment and informality further constrain ability to pay. Where affordability is the binding constraint, marginal quality differences – especially in a single service domain – may not shift enrolment. Secondly, choice sets differ across space. In the north, options are more numerous; proximity and convenience may dominate decision-making, dampening any incremental contribution of quality to enrolment decisions. In the south, where facility options are fewer, the observable quality of available facilities plausibly becomes more salient for households weighing the value of coverage. This heterogeneity suggests that quality weighs more heavily where density is low, while in higher-density contexts, convenience and choice play a greater role. Across both settings, however, socioeconomic factors – particularly wealth and education – remain the most consistent predictors of enrolment.

Our findings reveal how Namibia’s structural inequities shape these gradients. While ANC quality shows no national association with enrolment, the stark 5% versus 70% coverage gap between poorest or richest quintiles persists across all quality levels (Figure 5) – reflecting Namibia’s formal or informal divide where educated urbanites access employer-sponsored schemes such as PSEMAS, but the ≥ 60% informal workforce faces premiums consuming 22% of income. Education likely signals both financial literacy for scheme navigation and formal job access. This dominance of socioeconomic position over service quality underscores a core tension in Namibia’s dual economy: public facilities serve the structurally excluded, yet insurance remains stratified by formal employment and wealth.

Although our analysis focused on ANC as a proxy for maternal healthcare quality, it represents only one dimension of service delivery. ANC provides contemporaneous, measurable indicators of structure, process and client experience, offering a consistent window into the service environment most directly relevant to surveyed women. The null national association should therefore be interpreted as evidence that improvements in this slice of quality do not, on average, coincide with higher insurance uptake – though they may do so in contexts where facility choice is limited.

### Policy implications

Three policy directions follow. The most robust predictors of enrolment are wealth and education. Expanding coverage will likely require pro-poor financing (sliding-scale premiums, targeted subsidies or vouchers and reduced cost sharing for the poor) and simplified enrolment procedures. Without affordability reforms, uptake gains among the lowest quintiles are likely to be limited, regardless of quality improvements. Education’s strong association with enrolment underscores the need for communication strategies that improve understanding of the importance of insurance as a risk management tool, benefits packages, eligibility and claims processes. Quality investments should continue for intrinsic reasons (health outcomes, dignity and safety). The south–north contrast suggests that targeted quality upgrades could matter for enrolment where facility choice is limited; nationally, however, quality improvements alone are unlikely to improve enrolment without affordability and educational efforts.

Recent policy developments reinforce these points. The government’s directive that senior officials use public facilities, paired with planned investments in staffing, medicines and infrastructure is intended to boost confidence in the public sector.^42^ Such measures are more likely to support insurance expansion if they are coupled with concrete affordability improvements for households and clear communication about entitlements. In practice, this implies aligning quality upgrades with reforms to premium structures and enrolment processes, so that households perceive both better services and a financially workable path into coverage. Quality, financing and enrolment design must advance together for expanded coverage to translate into meaningful financial protection and use of needed services.

Policy roadmap from our findings: Firstly, close the 5% – 70% coverage gap with income-indexed premium subsidies targeting Q1–Q2 households and community health worker enrolment drives in informal settlements. Secondly, pair these with targeted southern quality upgrades where our models show quality’s strongest link. Thirdly, leverage education gradient with health facility-based insurance counselling during ANC visits (where 100% attendance provides a teachable moment). This sequencing recognises that socioeconomic barriers dwarf quality nationally, positioning quality investments to amplify – rather than substitute for – affordability reforms.

This interpretation resonates with the synthesis provided by Das and Do, who reviewed the landscape of health insurance in LMICs over the past three decades. Their analysis highlights that persistently low demand for insurance is less about technical supply-side constraints – such as equipment shortages or weak clinical processes – and more about systemic demand-side barriers, including adverse selection, administrative burdens and households’ limited perception of value.^72^ Namibia’s experience fits within this broader pattern: while quality remains vital for health outcomes and trust, it is affordability, information and broader socioeconomic conditions that appear to shape voluntary enrolment decisions most strongly.

This analysis demonstrates the feasibility and value of geospatially linking a facility census to household clusters to characterise local service quality at the national scale. Using buffers, inverse distance weighting and the use of a facility census address, in part, the well-documented misclassification risk from DHS cluster displacement and avoid the pitfalls of ‘nearest facility’ linkage. The approach is transferable to other LMICs with SPA and/or SARA-type audits and DHS-style surveys, enabling comparable investigations of quality–demand relationships across settings.

### Limitations

Several limitations should be noted. The SPA’s observation module may be susceptible to Hawthorne effects^73^; a weighted, multidimensional index improves on perception-only measures but cannot capture all facets of quality. Assigning cluster-level scores masks within-buffer variation and potential bypassing of facilities. Although buffers and inverse distance weighting reduce error from DHS displacement, residual misclassification is possible. The analysis focuses on ANC; household insurance decisions may reflect broader domains (child health, non-communicable diseases care, emergency and surgical services). The SPA precedes the DHS, but interim reforms may have altered quality; if so, estimates may understate any true association.^74,75^ We did not incorporate explicit time-varying regional indicators of health expenditure or facility upgrades because comparable, consistently measured data at the appropriate spatial scale were not available, and simple proxies risked introducing additional measurement error. More broadly, the 4-year gap means that quality is measured with some temporal error, which would be expected to bias estimates towards finding weaker rather than stronger relationships. As a cross-sectional study, our estimates cannot be interpreted as strictly causal. Temporal ordering (SPA preceding DHS) reduces concerns of reverse causality, and robustness across multiple specifications (including buffer sizes, quintiles, urban-only models and urban–rural interactions) strengthens confidence in the observed patterns. Nonetheless, unobserved confounding remains possible.

### Directions for future research

Future research should move beyond ANC to develop multi-service quality indices encompassing other areas of healthcare. It should also combine geospatial linkage with longitudinal or panel designs to strengthen causal inference, incorporate qualitative evidence on how households perceive quality and weigh it against price and convenience in enrolment decisions and undertake cross-country comparisons to assess whether the observed pattern – quality’s limited role nationally but greater salience where options are few – generalises beyond Namibia.

## Conclusion

Improving the quality of care is essential for strengthening health outcomes within health systems; however, on its own, it is unlikely to drive health insurance uptake at the national level in Namibia. While modest associations between quality and enrolment are observed in underserved regions with lower facility density, the overall findings indicate that socioeconomic inequalities—particularly differences in wealth and education—remain the primary determinants of insurance participation. These results suggest that decisions to enroll in health insurance are shaped less by marginal improvements in service quality and more by households’ ability to afford coverage and their level of knowledge about insurance. Consequently, efforts to expand insurance coverage should prioritize reducing financial barriers through targeted subsidies and pro-poor financing mechanisms, alongside efforts to improve knowledge of health insurance and its potential benefits. At the same time, continued investments in quality of care remain critical, particularly in underserved areas where quality may play a more influential role in shaping demand. Taken together, these findings underscore the need for integrated policy approaches that align quality improvements with affordability and access to achieve more equitable progress towards universal health coverage.
